# Institutional Biobanking as shared public health infrastructure: a financially sustainable service center model

**DOI:** 10.3389/frhs.2026.1778446

**Published:** 2026-03-18

**Authors:** M. V. Olson

**Affiliations:** The Johns Hopkins BioBank, Genetic Resources Core Facility, Department of Genetic Medicine, Johns Hopkins University School of Medicine, Baltimore, MD, United States

**Keywords:** biospecimen management, core facility, financial stability, institutional biobanking, public health infrastructure, quality management, service center, sustainability

## Abstract

Institutional biobanks are essential infrastructure supporting clinical research, translational discovery, and public health preparedness. However, many biobanks rely on institutional subsidies or unstable grant funding, creating long-term financial vulnerability. The Johns Hopkins Biobank, operating within the multi-division Genetic Resources Core Facility (GRCF), provides a practice-based example of a financially resilient model for academic biobanking. As a College of American Pathologists (CAP) accredited program structured as a fee-for-service enterprise, the Biobank targets a net-zero financial balance in accordance with U.S. federal guidance for service centers. All operational costs, including personnel, infrastructure, and institutional overhead, are recovered through transparent annual storage fees and transactional service charges, enabling high-quality stewardship without reliance on external subsidy. Integration within a broader service center portfolio provides operational flexibility, supports rigorous governance and regulatory compliance, and ensures continuity during periods of fluctuating research demand. This case illustrates how aligning biobanking with institutional service center frameworks can strengthen financial sustainability, enhance accountability, and support long-term access to high-quality biospecimens as part of public health research infrastructure. This model supports public health preparedness by ensuring stable access to biospecimens critical for infectious disease response, clinical trials, and translational science. Further, it offers transferable lessons for academic institutions seeking to future-proof biobanking operations while upholding rigorous standards for quality and participant trust.

## Introduction

Biobanks are foundational components of modern biomedical research and precision health, ensuring that high-quality biospecimens remain available for clinical studies, translational discovery, and public-health preparedness (1–4). As repositories have grown in scope and strategic importance, academic institutions face rising expectations to ensure long-term continuity of these critical infrastructures while maintaining rigorous governance, data protection, and ethical oversight.

Despite their importance, many institutional biobanks operate in financially vulnerable positions. Biospecimen stewardship requires continuous investment in specialized personnel, infrastructure, regulatory compliance, and emergency response systems. When these costs outpace institutional subsidies or fluctuating grant support, research programs and clinical readiness may be placed at risk (5). In this context, sustainability refers not only to environmental responsibility, but fundamentally to organizational and financial resilience, the ability to remain operational and effective during shifting economic and scientific climates (6). As biospecimen infrastructure underpins equitable research access, institutional models that embed accountability and resource governance are increasingly recognized as essential public-health policy tools.

This perspective describes the Johns Hopkins Genetic Resources Core Facility (GRCF) Biobank as a model of financially resilient institutional biobanking. Established in 1989 and operating under College of American Pathologists (CAP) accreditation (7), the Biobank is fully embedded within a multidisciplinary service center framework that targets net-zero operations. Through a fee-for-service structure aligned with NIH service center principles, the GRCF Biobank recovers ongoing costs while maintaining stringent standards for access control, traceability, and continuity planning.

Rather than relying on episodic institutional support, the GRCF Biobank functions as a stable infrastructure resource for public-health–relevant research, with its financial structure enabling sustained operational quality, risk mitigation, and capacity for strategic growth. This model offers a transferable framework for institutions seeking to preserve high-quality biobanking capabilities without jeopardizing long-term viability.

## Operational model: A service center approach to institutional Biobanking

**Figure 1:**
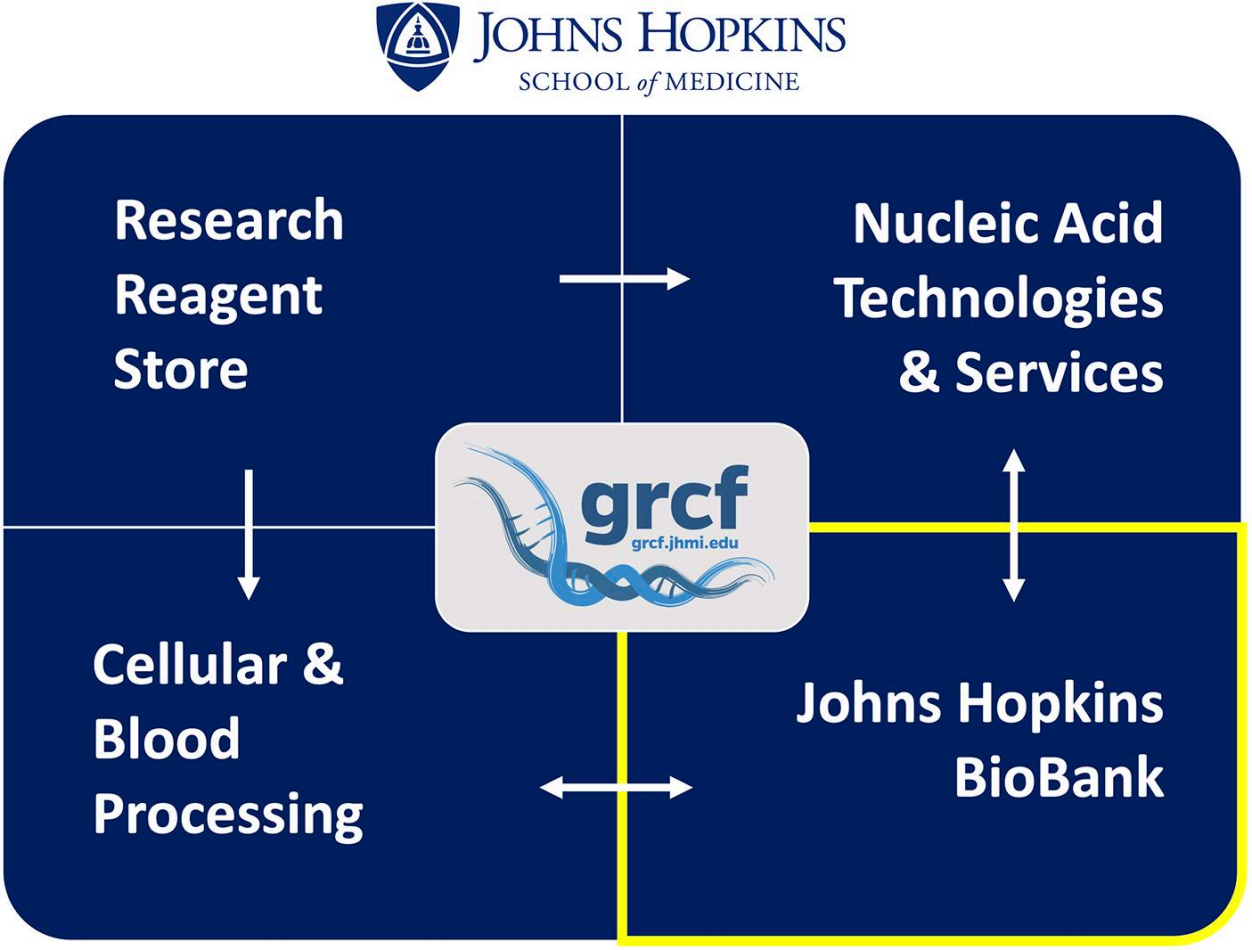
The GRCF service center model. The Genetic Resources Core Facility (GRCF) comprises four integrated divisions—Research Reagent Store, Nucleic Acid Technologies & Services, Cellular & Blood Processing, and the Johns Hopkins Biobank—linked through coordinated workflows that streamline project support from procurement to long-term specimen storage.

The Johns Hopkins Genetic Resources Core Facility (GRCF) Biobank operates within a multi-division service center that provides institution-wide research infrastructure. The GRCF includes four interconnected divisions: a reagent store, nucleic acid services, cell and blood processing, and the Biobank, functioning together under a targeted net-zero financial model (Figure 1). The Biobank supports both prospective clinical trials and long-term research repositories, offering stewardship for both investigator-initiated and institutionally strategic collections. Although the repository supports a range of biospecimen types, the collection is predominantly human, reflecting its central role in clinical and translational research infrastructure. All costs, including personnel and overhead, are recovered through user fees consistent with NIH guidance for research service centers.

This integrated structure delivers two major advantages. First, the Biobank can scale operations to meet institutional demand without dependence on central subsidy or external funding mechanisms. Second, close alignment with service-based laboratory workflows ensures that biospecimens can move seamlessly from acquisition to processing, analysis, and long-term preservation. As scientific priorities evolve, the multi-division model provides financial and operational buffering: surpluses and shortfalls are distributed across shared infrastructure rather than destabilizing a single biobanking unit.

To support cost transparency and predictability for users, services are recovered through annual storage fees and transaction-based charges for retrievals, shipments, and processing tasks. Importantly, the Biobank maintains CAP Biorepository accreditation, ensuring oversight of ethical compliance, access controls, and rigor in specimen handling. This structure preserves the autonomy of principal investigators, who retain full scientific ownership, while enabling institution-wide governance of infrastructure, risk, and quality.

Through this service center framework, the Biobank acts not only as a storage solution but as a public-health-critical facility: providing sustainable access to high-quality biospecimens while strengthening institutional resilience, continuity planning, and research capacity.

## Governance as a financial accountability mechanism

Financially resilient biobanking requires governance structures that ensure infrastructure is aligned with institutional priorities, regulatory standards, and scientific value (Table 1). Within the Johns Hopkins model, governance serves as a resource-allocation tool**,** helping the institution make informed decisions about which biospecimen collections merit long-term preservation and what level of infrastructure investment they require.

**Table 1:** Comparison of biobanking models.

Feature	Stand-alone institutional biobank	GRCF biobank service-center model
Funding Base	Institutional subsidy, grants, philanthropy	User fees (storage + actionable requests)
Financial Stability	Vulnerable to budget cuts, grant cycles	Net-zero target; shortfalls buffered by GRCF
Governance	Often project- or department-specific	CAP-accredited, centralized governance
Access	May be limited or slow	Restricted but responsive (≤4 h retrieval)
Integration	Independent or siloed	Embedded in multi-division core facility
Sustainability Outlook	Dependent on continued institutional support	Sustainable, scalable, replicable

Stand-alone biobanks typically rely on subsidies or grants and face financial instability, while the GRCF Biobank service center model achieves sustainability through fee-for-service operations, CAP accreditation, and integration within a multi-division.

All materials stored within the GRCF Biobank remain under the scientific and ethical authority of the originating investigators. The Biobank serves as steward of the physical specimens and associated inventory metadata required for traceability, regulatory compliance, and chain-of-custody oversight. Ownership of biospecimens, as well as any research or clinical data generated from their use, remains with the originating principal investigators under their respective IRB approvals and consent frameworks. However, transfer into the centralized facility requires basic documentation of regulatory compliance, inventory traceability, and continued research relevance. These expectations help eliminate unnecessary long-term storage of orphaned, undocumented, or scientifically obsolete assets, an important financial safeguard for the institution.

Integration within a broader service center structure also embeds cost transparency into stewardship. Annual storage fees reflect actual institutional costs, including facility infrastructure, personnel, and continuous monitoring. These charges prompt principal investigators to periodically re-evaluate the value and volume of their collections, supporting proactive disposition planning and preventing unchecked growth of dormant material. In parallel, fee-for-service structure provides accountability for newly requested storage, ensuring that expansion is based on demonstrated scientific need rather than convenience or legacy practice.

Governance policies are further reinforced through consultative purchasing oversight, requiring researchers to evaluate centralized storage before acquiring new equipment. This approach reduces duplication, mitigates unplanned risk associated with decentralized storage, and ensures all new additions to institutional infrastructure align with long-term sustainability and continuity standards.

By linking access, documentation, and charges to stewardship expectations, the GRCF Biobank governance structure functions as a financial control system, one designed not to restrict research, but to ensure that high-quality biobanking is preserved for the projects and populations that depend on it.

## Operational resilience and continuity planning

Biobanks serve as long-term custodians of irreplaceable research materials, assets that are often tied to clinical trials, longitudinal cohorts, or consent-dependent community partnerships. The GRCF Biobank's financial model is therefore closely integrated with continuity planning**,** ensuring that day-to-day financial decisions strengthen, not jeopardize, future specimen access.

Centralized monitoring, redundant storage systems, and secure access controls are foundational elements of the model. By consolidating institutional risk into a professionally managed facility, the Biobank reduces exposure to unexpected personnel turnover, equipment failures, or disruption within individual laboratories. Twenty-four-hour emergency response support and validated transfer workflows enable rapid protection of materials from decentralized freezers during outages or unplanned events.

Continuity planning extends beyond equipment reliability to organizational resilience. Because the Biobank operates within a diversified service center, temporary demand fluctuations in one division can be absorbed without compromising storage quality or staffing resources. In this way, the financial structure reinforces operational stability, ensuring that biobanking functions remain uninterrupted regardless of short-term funding variability or shifts in scientific priorities.

## Integrated service delivery strengthens institutional value

Beyond long-term preservation, the GRCF Biobank plays an active role in the research lifecycle by providing a centralized interface between clinical sample acquisition, laboratory processing, and downstream analysis. As a division within a comprehensive service center, the Biobank coordinates with companion units to deliver end-to-end infrastructure, from initial patient contact or specimen generation through nucleic acid preparation, data generation, and archival storage.

This integrated framework improves efficiency and reduces administrative burden for laboratories that would otherwise navigate multiple, disconnected service providers. It also expands access to biobanking for investigators who lack the personnel or operational capacity to manage freezer systems, sample movement, or regulatory documentation independently.

By aligning biospecimen stewardship with institutional workflows, the Biobank enhances research readiness, supporting faster project initiation, standardized quality practices, and cross-departmental collaboration. These institutional benefits strengthen the case for continued investment in centralized biobanking, even within an unfunded, net-zero service model.

## Economic stewardship principles for sustainable biobanking

A core function of the GRCF Biobank's financial model is transparent accountability. Pricing structures are based on predictable annual storage fees and defined service transactions, allowing investigators to budget prospectively rather than rely on unpredictable internal subsidies. Fee schedules are recalculated annually using NIH service center principles to ensure full cost recovery for staffing, consumables, storage infrastructure, and oversight activities.

This economic structure is intentionally designed to promote responsible stewardship**.** Investigators routinely reevaluate holdings to preserve only scientifically necessary materials, reducing long-term waste and administrative burden. At the institutional level, consolidated procurement and shared infrastructure eliminate duplicative resources that would otherwise diffuse operational costs.

By treating biospecimen preservation as a managed institutional service instead of an unfunded laboratory responsibility, the Biobank positions cost transparency as a public-health-aligned strategy: ensuring access for current research needs while safeguarding the future availability of critical materials.

## Community engagement and institutional accountability

Sustained success of a centralized biobank requires cultural alignment between service providers and the research community. To support this alignment, the GRCF Biobank incorporates structured engagement mechanisms, including biennial user surveys, advisory input through institutional leadership, and proactive consultation during new study planning.

Investigators retain scientific autonomy, ownership of specimens, and control of associated clinical data. All stored biospecimens remain governed under their original informed consent and IRB approvals, and the Biobank does not assume ownership of clinical data. The Biobank's role is stewardship, not scientific direction, ensuring traceability, consent compliance, and secure access while respecting the primacy of investigator governance.

This collaborative approach builds trust, improves transparency in how specimens are managed, and positions the biobank as a shared institutional asset. It also ensures that operational changes, such as workflow optimization or policy modifications, reflect the needs and realities of the research community.

## High-Level outcomes and institutional learnings

The GRCF Biobank has operated under a service center financial structure for more than a decade and under CAP Biorepository Accreditation since 2013. During this time, operations have been sustained entirely through fee-for-service revenue, with rate schedules recalculated annually to maintain a net-zero balance without institutional subsidy. This financial consistency has enabled uninterrupted support for research across the Johns Hopkins enterprise, even during periods of constrained federal and foundation funding.

The Biobank currently supports more than 400 IRB-approved studies and manages a dynamic cohort collection that fluctuates with investigator needs, averaging approximately 1,800 service requests per year through the combined activities of accessioning, retrieval, processing, and distribution. Integration within a multi-division service center allows for coordinated workflows and shared staffing expertise—including logistics specialists, compliance oversight, and customer support—that individual biobanks typically must support independently.

Through this institutional model, several practical lessons emerged:

1. Centralized coordination enables scale without proliferating local infrastructure.
2. Financial transparency strengthens user trust, improving payment compliance and forecasting.
3. Integrated services encourage scientific collaboration and cohort reuse, reducing redundant research spending.
4. Institutional alignment protects continuity**,** ensuring biobanking remains stable as research priorities evolve.

These outcomes show that financial resilience can be achieved not only through cost control, but through organizational design that distributes responsibility and value creation across the research ecosystem. Ongoing evaluation with institutional leadership continues to refine the model as research technologies and financial pressures change.

## Conclusion

The Johns Hopkins GRCF Biobank demonstrates that institutional biobanking can remain financially stable without sacrificing scientific rigor, governance, or equitable access. Embedding the biobank within a diversified service center structure provides operational flexibility, distributes financial responsibility, and connects specimen preservation to the broader research ecosystem it supports. Transparent pricing, standardized stewardship expectations, and centralized expertise allow the Biobank to withstand variable demand while meeting high-quality and regulatory standards.

Rather than positioning financial sustainability and research excellence as competing objectives, this model illustrates that public-health-aligned infrastructure can deliver both. By integrating biobanking into an institutional system built on accountability, responsiveness, and shared resources, academic organizations can enhance scientific continuity and reduce vulnerability to funding instability. Although each institution must tailor implementation to its local environment, the principles described—service center alignment, fee-for-service transparency, and community-guided governance—offer a transferable strategy for strengthening the financial resilience of research infrastructure. Like all institutional models, this approach is shaped by the regulatory and financial structures of its local environment. Institutions with different subsidy models, cost governance, or national funding policies may need to adapt specific financial levers. However, the underlying principles of accountability, transparency, and integrated stewardship remain broadly transferable. As institutions continue to build research preparedness capacity worldwide, models like this may inform national strategies for sustainable biobanking infrastructure.

## Data Availability

The original contributions presented in the study are included in the article/Supplementary Material, further inquiries can be directed to the corresponding author.
